# Antibiotic prescriptions for oral diseases in India: evidence from national prescription data

**DOI:** 10.1186/s12903-023-02889-0

**Published:** 2023-03-25

**Authors:** Manu Raj Mathur, Deepti Nagrath, Vijay Kumar Mishra, Rebecca Harris, Syed Saif Saeed, Sakthivel Selvaraj, Aashna Mehta, Habib Hasan Farooqui

**Affiliations:** 1 Queen Mary University of London, Mile End Rd, Bethnal Green, London, E1 4NS England; 2 Public Health Foundation of India, Plot No. 47 Sector 44, Institutional Area, Gurgaon, 122002 Haryana India; 3 University of Liverpool, Liverpool, L69 3BX UK; 4 University College London, Gower St, London, WC1E 6BT UK; 5 Health Economics, Financing and Policy, Public Health Foundation of India, Plot No. 47 Sector 44, Institutional Area, Gurgaon, 122002 Haryana India; 6 College of Medicine, QU Health, Qatar University, Doha, Qatar

**Keywords:** Antimicrobial Resistance, India, Dental Ailments, Prescription Rates

## Abstract

**Introduction:**

The key objective of this research was to describe the prescription rate of various antibiotics for dental problems in India and to study the relevance of the prescriptions by analysing antibiotic types associated with different dental diagnoses, using a large-scale nationally representative dataset.

**Methods:**

We used a 12-month period (May 2015 to April 2016) medical audit dataset from IQVIA (formerly IMS Health). We coded the dental diagnosis provided in the medical audit data to the International Statistical Classification of Diseases and Related Health Problems (ICD-11) and the prescribed antibiotics for the diagnosis to the Anatomic Therapeutic Chemical (ATC) -2020 classification of the World Health Organization. The primary outcome measure was the medicine prescription rate per 1,000 persons per year (PRPY_1000_).

**Results:**

Our main findings were—403 prescriptions per 1,000 persons per year in the year 2015 -2016 for all dental ailments. Across all ATC level 1 classification, ‘Diseases of hard tissues’ made up the majority of the prescriptions. ‘Beta-lactam’, ‘Penicillin,’ and ‘Cephalosporins’ were the most commonly prescribed antibiotics for dental diagnoses followed by ‘Macrolides’ and ‘Quinolones’. ‘Dental caries’, ‘Discoloration of tooth’, and ‘Toothache’ were the most common reasons for ‘Beta-Lactams’ and ‘Penicillin’ prescriptions.

**Conclusion:**

To conclude our study reports first ever country (India) level estimates of antibiotic prescription by antibiotic classes, age groups, and ICD-11 classification for dental ailments.

**Supplementary Information:**

The online version contains supplementary material available at 10.1186/s12903-023-02889-0.

## Introduction

Although modern medicine with its discovery of drugs [1] and other technological advances has been hailed for significantly reducing mortality and morbidity worldwide, the problem of Antimicrobial Resistance (AMR) has emerged as one of the principal public health problems of the 21^st^ century [2]. A growing challenge for health systems across the world is the “*Rational Use of Drugs”* which is defined as prescribing an appropriate dose of medicines according to a patient’s clinical diagnosis, well-being, amenability and cost [3]. In the Nairobi Declaration of 1985 [4] rational drug use was identified as a global issue, relevant to both developing and developed countries. In developed countries, health professionals in many countries face the issues of selecting from a multitude of drugs available together with a tidal wave of information and having to deal with commercial influences from drug promotions. The rational use of drugs has also been discussed by the World Health Organization through ‘’*The International Network for Rational Use of Drugs* (INRUD) [5] in 1989; *“Policy Perspectives on Medicines”* (WHO 2002) in 2002; and during the 60th World Health Assembly in 2007 [6].

Dental providers are not immune to the irrational prescription of drugs including antibiotics [7–10]. India is considered to be a major consumer of antibiotics in the world, and this appears to be expanding further [11]. Although several studies highlight the prescription practices of doctors in the India [12–16], studies on dental providers are few, involving either interview data or small-scale surveys, and have limited generalisability across the country [17–19]. In an effort to address this gap, we, therefore, aim to describe the prescription rate of various antibiotics for dental problems in India and then to investigate the appropriateness of the prescriptions by analysing antibiotic types associated with a range of dental diagnoses, using a large-scale nationally representative dataset.

## Methods

### Data source and setting

We used a 12-month period (May 2015 to April 2016) medical audit dataset from IQVIA. The IQVIA’s prescription (outpatient medical audit) data were collected from a panel comprising 4600 health professionals from the private allopathic sector selected through a multi-stage stratified random sampling, including General Practitioners e.g. MBBS (Bachelor of Medicine Bachelor of Surgery), Non-MBBS General Practitioners, and other Medical Specialties (Dentists, Paediatricians, Gynaecologists, and others) from 23 metropolitan areas (population more than 1 million), 128 class 1 towns (population more than 100,000) and 1A towns (population less than 100,000) in India. These outpatient data were then extrapolated to reflect the prescription pattern of these professionals.

The prescription rates (age and gender-wise patterns) were calculated using age and gender-specific population (projected population) provided by the National Commission on Population, Government of India [20].

The prescription patterns for data relevant to dental diagnoses for the current analysis were extracted from the parent data set. The parent data consisted of prescription patterns of various diseases ( such as infectious and parasite diseases, diseases of the blood and blood-forming organs, endocrine, nutritional and metabolic diseases, diseases of the nervous system, diseases of the circulatory system, diseases of the musculoskeletal system, mental and behavioural disorders etc.). The current data set was extracted out of the parent data set with the specific diagnosis of diseases of mouth/teeth/tongue. The collected data was made free of health professionals’ identifiers (anonymised) thus inhibiting any linkage to an individual.

The IQVIA dataset contains 75 distinct dental diagnoses and 1196 drugs, including combinations of oral solids and liquids. We classified all the drugs prescribed by providers into 14 broad categories according to the Anatomical Therapeutic Chemical (ATC) 2020 [21] – Level 1 classification, given by World Health Organisation (WHO), while the remaining unidentified drugs and/or entities were treated as “Others” (Supplementary Table 1). We coded the dental diagnoses based on the International Statistical Classification of Diseases and Related Health Problems, (ICD-11) 11th Revision [22]. Any diagnoses in the dataset not included in the ICD-11 classification were grouped and labelled as “Not Defined” (Supplementary Table 2). The total number of prescriptions for all oral health conditions (75 dental diagnoses) considering 1196 drugs were 52,02,41,570.

The primary outcome measure was the medicine prescription rate per 1,000 persons per year (PRPY_1000_) that measures the annual utilisation of drugs. We then estimated age-specific, gender-specific, and disease-specific drug prescription patterns for the year 2015 -2016.

### Statistical analysis

The population estimates were obtained from the report of the technical group on population projections constituted by the National Commission on Population, Government of India [20]. Age groups were determined by the classification already provided in the medical audit data.

Prescription rate per 1000 persons per year was calculated as below:

$$PRPY1000=\left(\frac{n}{P}\right)*\frac{1000}{t}$$

Where, PRPY_1000_= Prescription rate per 1000 persons per year

n= number of prescriptions

P= population, t= number of years

All the statistical analyses were performed in STATA (version-13.0, Stata Corp LP) software, Parallel Edition [23].

## Results

The total number of prescriptions in the year 2015 -2016 for all dental ailments was 52,02,41,570 or 403 prescriptions per 1,000 persons per year. The drugs prescription rates for different age groups ‘30–39 years,’ ‘40–49 years,’ and’50–59 years’ were 53.9, 65.5, and 76.6 per 1,000 persons per year, respectively. The highest prescription rate was estimated for the age group ‘60–64 years’ (120.7 per 1,000 persons per year). The prescription rate for ‘males’ (407.8 per 1,000 persons per year) was found to be marginally higher than that of ‘females’ (398.4 per 1,000 persons per year), as shown in Table 1. The out-patient antibiotic prescription rate for dentists and/or general practitioners was estimated to be 89.2 per 1,000 persons per year (i.e. 22.1% for the given sample) for various dental diagnoses (Table 2).

**Table 1:** Percentage distribution of outpatient drugs prescriptions by patient demographic characteristics in India (2015–2016)

**Patient demographic characteristic**		**%**	**PRPY1000**^b^
Age-Group	**Total Prescriptions**^a^** (in millions)**		
0–4 Years	3.2	0.6	**2.3**
5–9 Years	14.2	2.7	**11.9**
10–19 Years	25.7	5.0	**10.2**
20–29 Years	93.1	17.9	**40.2**
30–39 Years	102.6	19.7	**53.9**
40–49 Years	100.7	19.4	**65.5**
50–59 Years	82.5	15.9	**76.6**
60–64 Years	46.4	8.9	**120.7**
65 + Years	51.8	10.0	**64.8**
Gender			
Female	249.7	48.0	**398.4**
Male	270.5	52.0	**407.8**
Total	**520.2**	**100**	**403.2**

^a^Moving Annual Total (MAT)—The total value of a variable, over the course of the previous 12 months

^b^Prescriptions Per 1000 Persons Per Year (PRPY)

**Table 2:** Percentage distribution of outpatient drugs prescriptions by Anatomical Therapeutic Chemical (ATC) classification in India (2015–2016)

**Drugs**	**Total Prescriptions**^a^	**%**	**PRPY 1000**^b^
A: Alimentary tract and metabolism	12,83,93,312	24.68	99.51
B: Blood and blood forming organs	33,06,814	0.64	2.56
C: Cardiovascular system	1,20,301	0.02	0.09
D: Dermatological	9,05,701	0.17	0.70
G:Genito urinary system and sex hormones	2,03,863	0.04	0.16
H:Systemic hormonal preparations excluding sex hormones and insulins	30,79,992	0.59	2.39
J:Anti-infectives for systemic use	11,51,04,511	22.13	89.21
L:Antineoplastic and immunomodulating agents	4,020	0.00	0.00
M:Musculo skeletal system	13,67,02,976	26.28	105.95
N:Nervous system	2,59,01,869	4.98	20.08
P:Antiparasitic products insecticides and repellents	1,63,38,346	3.14	12.66
R:Respiratory system	14,04,604	0.27	1.09
S:Sensory organs	97,256	0.02	0.08
V:Various	48,52,824	0.93	3.76
Not defined	8,38,25,181	16.11	64.97
Total	**52,02,41,570**	**100**	403.21

^a^Moving Annual Total (MAT)—The total value of a variable, over the course of the previous 12 months

^b^Prescriptions Per 1000 Persons Per Year (PRPY 1000)

**Fig. 1:**
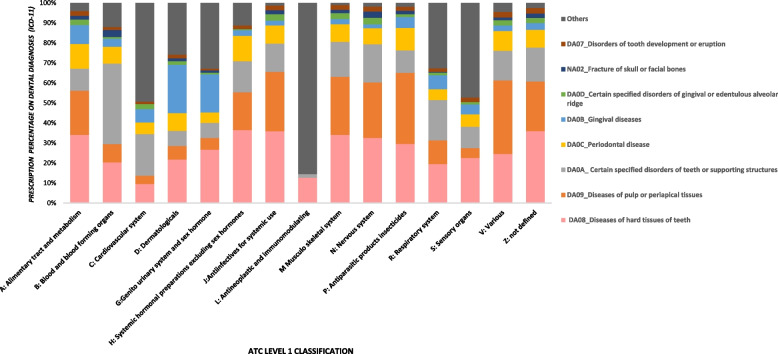
Percentage distribution of diagnosis (ICD 11) by Anatomical Therapeutic Chemical (ATC) Classification

Across all ATC level 1 classification, ‘Diseases of hard tissues’ made up the majority of the prescriptions. For antibiotics (J), the highest prescriptions were dispensed for ‘Disease of hard tissues of teeth’ (39.9%), ‘Disease of Pulp or periapical tissues’ (26.6%), ‘Certain specified orders of teeth and support structures’ (14%) and ‘Periodontal diseases’ (9.2%). A significant percentage of ‘Systemic hormonal preparations excluding sex hormones and insulins’ (H) was prescribed for ‘Diseases of the hard tissues’ (36.5). Similarly, medications for ‘Blood and Blood forming organs’ (B) were prescribed for ‘Certain specified disorders of teeth or supporting structures’ (40.25%) as shown in Fig. 1.

‘Beta-Lactams’ and ‘Penicillin’ were particularly used for the categories ‘Diseases of hard tissues of teeth’ (60.4%) and ‘Disorders of tooth development or eruption’ (61.4%), shown in (Table 3).

**Table 3:** Percentage distribution of diagnosis (ICD11) by ATC (J: Anti-infective for systemic use) classification, (2015–2016)

	J:Antiinfectives for systemic use									
ICD-11 Classification	**Tetracycline**	**Amphenicols**	**Beta-Lactams, Penicillin**	**Beta-Lactams, Cephalosporins**	**Sulfonamides & Trimethoprim**	**Macrolides & Lincosamides**	**Aminoglycosides**	**Quinolones**	**Others**	**Total**
Diseases of hard tissues of teeth	2.70	0.00	60.49	25.32	0.06	2.65	0.00	0.05	8.74	**4,13,81,333**
Diseases of pulp or periapical tissues	2.65	0.00	55.37	32.78	0.24	4.10	0.00	0.05	4.81	**3,40,79,476**
Certain specified disorders of teeth or supporting structures	3.12	0.00	58.66	27.33	0.18	2.89	0.00	0.14	7.68	**1,62,05,079**
Gingival diseases	16.71	0.01	46.58	24.85	0.77	4.58	0.00	0.02	6.48	**26,56,844**
Periodontal disease	10.74	0.00	51.10	26.64	0.29	4.09	0.00	0.15	6.98	**1,06,08,482**
Certain specified disorders of gingival or edentulous alveolar ridge	1.38	0.00	59.02	32.18	0.00	3.62	0.00	0.01	3.78	**37,34,848**
Fracture of skull or facial bones	1.16	0.00	49.62	22.23	0.01	4.57	0.00	0.08	22.32	**23,14,386**
Disorders of tooth development or eruption	2.54	0.00	61.46	28.83	0.02	2.35	0.00	0.07	4.74	**26,67,485**
Others	6.33	0.06	32.12	39.65	0.58	8.47	0.06	0.62	12.11	**14,56,578**
Total	**43,45,775**	**3,048**	**6,55,24,003**	**3,26,26,556**	**1,95,952**	**39,46,464**	**952**	**91,038**	**83,70,723**	**11,51,04,511**

Whereas ‘Beta-lactam’, ‘Penicillin,’ and ‘Cephalosporins’ were the most commonly prescribed antibiotics for dental diagnoses followed by ‘Macrolides’ and ‘Quinolones’ (Table 4). On the other hand, the antimicrobials ‘Beta-Lactams’ and ‘Cephalosporins’ were the most commonly used prescriptions for ‘Diseases of pulp or periapical tissues’ (32.7%) (Table 3). ‘Tetracyclines’ were also found to be prescribed for ‘Disorders of tooth development or eruption’ (2.5%), ‘Discoloration of teeth’ (12.8%), and 'Abrasion’ (7.5%), as shown in (Tables 3 and 4). A more detailed breakdown of larger categories of dental diagnosis showed that ‘Dental caries’ (60.3%), ‘Discoloration of tooth’ (61.2), and ‘Toothache’ (51.0%), were the most common reasons for ‘Beta-Lactams’ and ‘Penicillin’ prescriptions.

**Table 4:** Percentage distribution of antibiotic prescriptions by top 15 dental diagnoses in India (2015–2016)

Dental Diagnoses	Tetracycline	Amphenicols	Beta-Lactams, Penicillin	Beta-Lactams, Cephalosporins	Sulphonamides & Trimethoprim	Macrolides and Lincosamides	Aminoglycosides	Quinolones	Others	Total
Abrasion of teeth	7.45	0.00	58.09	28.36	0.00	2.51	0.00	0.03	3.56	1,179,688
Dental abscess	2.35	0.00	54.16	34.13	0.17	3.66	0.00	0.05	5.47	11,407,230
Gingivitis	17.43	0.01	45.91	24.89	0.76	4.57	0.00	0.02	6.41	2,740,088
Periodontitis	7.76	0.00	54.77	27.25	0.21	3.70	0.00	0.11	6.21	15,260,442
Pulpitis	2.00	0.00	58.16	32.39	0.01	3.93	0.00	0.00	3.51	8,316,332
Others	6.21	0.04	39.59	37.25	0.28	5.41	0.01	0.48	10.74	2,077,683
Root canal	2.89	0.00	57.27	30.61	0.35	4.01	0.00	0.07	4.80	18,555,967
Dental caries	2.01	0.00	60.31	25.45	0.06	2.74	0.00	0.05	9.38	34,840,642
Oral prophylaxis	17.16	0.00	50.21	20.34	0.09	4.06	0.00	0.19	7.96	1,097,873
Fracture of tooth	1.16	0.00	49.62	22.23	0.01	4.57	0.00	0.08	22.32	2,314,386
Extraction	2.69	0.00	59.74	27.24	0.13	3.15	0.00	0.14	6.91	13,495,698
OSMF/ leucoplakia	7.76	0.00	29.16	39.44	1.24	9.66	0.29	0.45	12.01	234,041
Toothache	4.61	0.01	50.99	31.27	0.39	2.04	0.00	0.04	10.66	2,955,803
Discoloration tooth	12.79	0.00	61.15	17.76	0.05	2.42	0.00	0.00	5.83	442,086
Loss of teeth	2.92	0.00	73.47	19.72	0.13	2.41	0.00	0.00	1.34	186,552
Total	**4,345,775**	**3,048**	**65,524,003**	**32,626,556**	**195,952**	**3,946,464**	**952**	**91,038**	**8,370,723**	**115,104,511**

## Discussion

To the best of our knowledge, this is the first study using a nationally representative dataset to evaluate the estimates of outpatient antibiotic prescription rates for dental ailments and patterns with antibiotic prescription rates across age groups by diagnosis (ICD-11 classification) and antibiotic classification (ATC classification) in India. Our estimated antibiotic prescription rate for dental ailments was approximated to be 89.21 prescriptions per 1,000 persons per year which is much higher than the prescription rate of 77.5 prescriptions per 1,000 persons for the United States [24]. The results also suggest that most antibiotics prescribed for infection prophylaxis in dental ailments in India are irrational – a finding consistent with findings from other countries [8, 25].

The overall prescription rate of antibiotics for any ailment in India was estimated to be 412 prescriptions per 1,000 persons per year, in a previous study published in 2019 using the same dataset [11]. The estimates are much less compared to the UK (555 prescriptions per 1,000 persons) [26] and Greece (1,100 antibiotics per 1000 person) [27].

Excessive prescription of antibiotics for dental ailments in India is problematic for several reasons. Firstly, most oral health conditions commonly leading to pain, abscess formation, and/or tooth loss [28] can be successfully treated with clinical intervention rather than antibiotics [7, 10, 29]. Secondly, most practitioners do not know the type of micro-organism present in the suppuration, so their prescriptions lack specificity and are based on assumptions [8]. The situation regarding irrational use of antibiotics in India is not usual, as shown in previous reviews showing dentists prescribe a wide variety of antibiotics for various clinical and non-clinical indications [9, 30]. Our study also shows that Indian practitioners commonly prescribe antibiotics such as Tetracyclines (J01A) for disorders of tooth development or eruption, which can accumulate in the developing teeth and bone, with side effects such as discolouration of teeth at an early age. Other side effects such as gastrointestinal, haematological, neurological, dermatological, and allergic reactions are also associated with such irrational antibiotic use.

Within national guidelines governing the use of antibiotics in India, oral health applications are not mentioned in any of the national guidelines such as the National Policy for Containment of Antimicrobial Resistance, Establishment of the National Programme on AMR Containment under the Twelfth Five Year Plan (2012–2017) [31–33], National Action Plan for Anti-Microbial Resistance in India and the Treatment Guidelines for Antimicrobial Use in Common Syndromes (ICMR) [34].

There is a lack of comprehensive standard treatment prescription guidelines for oral diseases in India. The current standard treatment guideline for oral health in India was last released in 2010 [33], which focuses on only the two most common dental ailments; Dental Caries and Periodontitis (Supplementary Table 3). These guidelines recommend that ‘Beta-lactam,’ its derivatives (Amoxicillin), and analgesics (Brufen and Paracetamol) are the antibiotics of choice for common dental ailments. The guideline, however, fails to elaborate on other dental conditions. Rationally, conditions like pulpitis with moderate or severe symptoms with/without acute periodontitis do not require antibiotic coverage [35]. However, in our study, 58.16% of prescriptions had Beta-Lactams, Penicillin for the said diagnosis. Similarly, diagnoses such as toothache, discoloration tooth, halitosis were also treated with Beta-Lactams, Penicillin (J01C) and Beta-Lactams, Cephalosporins (J01D).

To summarize, the current study identifies the current state of antibiotic prescribing practices among dental health professionals. The paper highlights the need to develop and train dental health professionals that displays a wider range of skills, better understanding of AMR, and greater attention to evidence base practices.

### Strengths and limitations

To the best of our knowledge, this is the first study to evaluate the estimates of outpatient antibiotic prescription rates for dental ailments and patterns with antibiotic prescription rates across age groups by diagnosis (ICD-11 classification) and antibiotic classification (ATC classification) in India. The data used in the presented study has some inherent limitations. The diagnosis available in the data set was not charted to ICD 11 classification. Therefore, manual coding based on available diagnosis may have led to certain inaccuracies in the allocation of codes. The data collected was based on the panel stockist’s data, with inherent limitations such as lack of motivation of recording the data and its validation. The data set provided set provided by the source agency IQVIA was also limited to specific variables analysed in the manuscript. The analysis had to rely on the projected population taken from the National Commission on Population to determine prescription rates. Using prescription rates also ignored many factors that play a role in the outcome, such as the underlying condition of the patient; how sick the patient was; and the length of time of the measurement. We also had limited information on whether the prescriptions for the data set “Oral Cavity” were explicitly written only by a Dental Practitioner and not by any other General Practitioner.

## Conclusion

Through our analysis of a nationally representative dataset, we have highlighted the irrational use of antibiotics for oral diseases in India. The impact of irrational use of antibiotics is constantly increasing the burden of the disease with antibiotic resistance, drug dependence, side effects, morbidity, mortality, and financial loss. We need to strengthen our health systems by developing evidence based standard treatment guidelines for oral diseases and build capacity of dental health professionals for adhering to these guidelines. Further research is also needed to understand the factors (both from the prescriber as well as the consumer perspective) that promote antibiotic prescriptions by dental health professionals for oral diseases and whether antibiotics are used mainly for urgent care or routine oral health care. Integrating oral health services in overall health systems has been the call for action in the recently global policy discourse and appropriate use of antibiotics for oral diseases is one firm step in this direction.

## Supplementary Information

**Additional file 1:** **Supplementary Table 1.** Anatomical Therapeutic Chemical (ATC) classification. **Supplementary Table 2.** international statistical classification of diseases and related health problems (ICD – 11). **Supplementary Table 3.** Drugs for the treatment of dental ailments: standard treatment guidelines in India. **Supplementary Table 4.** Number distribution of diagnosis (ICD11) by ATC (J: Anti-infective for systemic use) classification, (2015-2016). **Supplementary Table 5.** Number distribution of antibiotic prescriptions by top 15 dental diagnoses in India.

## Data Availability

The data that support the findings of this study are available from IQVIA repository, [https://www.iqvia.com/solutions/real-world-evidence/real-world-data-and-insights] but restrictions apply to the availability of this data set and can be accessed upon a reasonable request to the data owner. The cleaned variables used specifically for this study are available from the authors upon reasonable request.
