# Supplementary material for: Antibiotic prescriptions for oral diseases in India: evidence from national prescription data
Source: BMC Oral Health. 2023 Mar 25;23:170. doi: 10.1186/s12903-023-02889-0 (PMC10039494; doi:10.1186/s12903-023-02889-0)
Supplement: Supplementary file 1 — Additional file 1: Supplementary Table 1. Anatomical Therapeutic Chemical (ATC) classification. Supplementary Table 2. international statistical classification of diseases and related health problems (ICD – 11). Supplementary Table 3. Drugs for the treatment of dental ailments: standard treatment guidelines in India. Supplementary Table 4. Number distribution of diagnosis (ICD11) by ATC (J: Anti-infective for systemic use) classification, (2015-2016). Supplementary Table 5. Number distribution of antibiotic prescriptions by top 15 dental diagnoses in India. [file 12903_2023_2889_MOESM1_ESM.docx]

**Supplementary Table 1**

**Anatomical Therapeutic Chemical (ATC) Classification**

| A | Alimentary tract and metabolism |
| --- | --- |
| B | Blood and blood-forming organs |
| C | Cardiovascular system |
| D | Dermatologicals |
| G | Genito urinary system, and sex hormones |
| H | Systemic hormonal preparations excluding sex hormones and insulins |
| J | Anti-infective for systemic use |
| L | Antineoplastic and immunomodulating agents |
| M | Musculo skeletal system |
| N | Nervous system |
| P | Antiparasitic products insecticides and repellents |
| R | Respiratory system |
| S | Sensory organs |
| V | Various |

**Supplementary Table 2**

**International Statistical Classification of Diseases and Related Health Problems (ICD – 11)**

| **DA01** | **Disorders of oral mucosa** |
| --- | --- |
| **DA02** | Miscellaneous specified disorders of lips or oral mucosa |
| **DA03** | Diseases of the tongue |
| **DA04** | Diseases of salivary glands |
| **DA07** | Disorders of tooth development or eruption |
| **DA08** | Diseases of hard tissues of teeth |
| **DA09** | Diseases of pulp or periapical tissues |
| **DA0A** | Certain specified disorders of teeth or supporting structures |
| **DA0B** | Gingival diseases |
| **DA0C** | Periodontal diseases |
| **DA0D** | Certain specified disorders of gingival or edentulous alveolar ridge |
| **DA0E** | Dentofacial anomalies |
| **DA0F** | Sensory disturbances affecting orofacial complex |
| **LA30** | Structural developmental anomalies of teeth and periodontal tissues |
| **LA31** | Structural developmental anomalies of mouth or tongue |
| **MD80** | Symptoms and signs of orofacial complex |
| **NA02** | Fracture of skull and facial bones |
| **NA03** | Dislocation or strain or sprain of joints or ligaments of head |
| **NA** | Symptoms related to the upper gastrointestinal tract |

**Supplementary Table 3**

**Drugs for the Treatment of Dental Ailments: Standard Treatment Guidelines in India**

| STANDARD TREATMENT GUIDELINES FOR ORAL HEALTH | |
| --- | --- |
| **Dental Caries** | |
| Cap Amoxicillin | 30-40mg/kg/day - 8 hourly for 5 days |
| Tab Paracetamol | 500mg- 8 hourly for 5 days |
| Tab Brufen | 400 mg - 8 hourly for 5 days |
| **In- case of Swelling/Cellulites or Abscess** | |
| Cap Amoxicillin | 30-40mg/kg/day 8 hourly – 5 days |
| Tab Tinidazole | 500 mg – 12 hourly for 5 days |
| Tab Paracetamol | 500mg - 8 hourly for 5 days |
| Tab Brufen | 400 mg- 8 hourly for 5 days |
| **Periodontitis** | |
| Tab Ciprofloxacin and  Tab Tinidazole | 500 mg – 12 hourly for 5 days |

**Supplementary Table 4**: Number distribution of diagnosis (ICD11) by ATC (J: Anti-infective for systemic use) classification , (2015-2016)

| **J:Antiinfectives for systemic use** | **ICD-11 Classification** | | | | | | | | |  |
| --- | --- | --- | --- | --- | --- | --- | --- | --- | --- | --- |
|  | **DA08 Diseases of hard tissues of teeth** | **DA09Diseases of pulp or periapical tissues** | **DA0A Certain specified disorders of teeth or supporting structures** | **DA0BGingival diseases** | **DA0CPeriodontal disease** | **DA0DCertain specified disorders of gingival or edentulous alveolar ridge** | **NA02 Fracture of skull or facial bones** | **DA07Disorders of tooth development or eruption** | **Others** | **Total** |
| Tetracycline (J01A) | 11,16,254 | 9,01,636 | 5,05,980 | 4,43,907 | 11,39,426 | 51,706 | 26,951 | 67,661 | 92,254 | **43,45,775** |
| Amphenicols (J01B) | 1,315 | 223 | 296 | 238 | 144 | 0 | 0 | 0 | 832 | **3,048** |
| Beta-Lactams, Penicillin (J01C) | 2,50,29,587 | 1,88,69,828 | 95,05,590 | 12,37,427 | 54,21,455 | 22,04,458 | 11,48,426 | 16,39,383 | 4,67,849 | **6,55,24,003** |
| Beta-Lactams, Cephalosporins (J01D) | 1,04,75,793 | 1,11,72,474 | 44,29,010 | 6,60,305 | 28,26,221 | 12,01,956 | 5,14,425 | 7,68,911 | 5,77,461 | **3,26,26,556** |
| Sulfonamides & Trimethoprim (J01E) | 23,773 | 82,039 | 29,242 | 20,499 | 31,161 | 0 | 248 | 496 | 8,494 | **1,95,952** |
| Macrolides & Lincosamides (J01F) | 10,96,763 | 13,98,007 | 4,68,989 | 1,21,631 | 4,33,930 | 1,35,252 | 1,05,797 | 62,686 | 1,23,409 | **39,46,464** |
| Aminoglycosides (J01G) | 126 | 0 | 0 | 0 | 0 | 0 | 0 | 0 | 826 | **952** |
| Other_antibiotics (not defined) | 30,71,224 | 16,12,143 | 10,05,505 | 1,67,315 | 6,22,765 | 1,40,406 | 3,11,051 | 1,05,945 | 1,51,890 | **71,88,244** |
| Quinolones (J01M) | 21,480 | 17,746 | 22,076 | 588 | 15,808 | 369 | 1,964 | 2,000 | 9,007 | **91,038** |
| Other_antibiotics(J01N) | 0 | 0 | 1,263 | 0 | 0 | 0 | 0 | 0 | 324 | **1,587** |
| Other_antiinfectives (Includes J04, 05, 06, 07,08) | 5,45,018 | 25,380 | 2,37,128 | 4,934 | 1,17,572 | 701 | 2,05,524 | 20,403 | 24,232 | **11,80,892** |
| **Total** | **4,13,81,333** | **3,40,79,476** | **1,62,05,079** | **26,56,844** | **1,06,08,482** | **37,34,848** | **23,14,386** | **26,67,485** | **14,56,578** | **11,51,04,511** |

**Supplementary Table 5** : Number distribution of antibiotic prescriptions by top 15 dental diagnoses in India

| **Oral Diagnoses** | **Tetracycline (J01A)** | **Amphenicols (J01B)** | **Beta-Lactams, Penicillin (J01C)** | **Beta-Lactams, Cephalosporins (J01D)** | **Sulfonamides & Trimethoprim (J01E)** | **Macrolides &Lincosamides(J01F)** | **Aminoglycosides(J01G)** | **Quinolones (J01M)** | **Others** | **Total** |
| --- | --- | --- | --- | --- | --- | --- | --- | --- | --- | --- |
| **Abrasion of teeth** | 87,853 | 0 | 6,85,333 | 3,34,505 | 0 | 29,651 | 0 | 350 | 41,996 | 11,79,688 |
| **Dental abscess** | 2,68,544 | 108 | 61,78,008 | 38,92,974 | 19,259 | 4,18,051 | 0 | 6,144 | 6,24,142 | 1,14,07,230 |
| **Gingivitis** | 4,77,699 | 238 | 12,57,987 | 6,82,020 | 20,790 | 1,25,147 | 0 | 588 | 1,75,619 | 27,40,088 |
| **Periodontitis** | 11,83,753 | 144 | 83,57,632 | 41,57,768 | 31,895 | 5,64,125 | 0 | 17,466 | 9,47,659 | 1,52,60,442 |
| **Pulpitis** | 1,66,708 | 0 | 48,37,025 | 26,93,295 | 1,216 | 3,26,481 | 0 | 0 | 2,91,607 | 83,16,332 |
| **Root canal** | 5,37,040 | 115 | 1,06,27,468 | 56,79,549 | 64,195 | 7,44,049 | 0 | 12,955 | 8,90,596 | 1,85,55,967 |
| **Dental caries** | 7,00,964 | 1,315 | 2,10,13,579 | 88,65,903 | 19,720 | 9,52,977 | 126 | 18,115 | 32,67,943 | 3,48,40,642 |
| **Oral prophylaxis** | 1,88,378 | 0 | 5,51,285 | 2,23,307 | 953 | 44,531 | 0 | 2,035 | 87,384 | 10,97,873 |
| **Fracture of tooth** | 26,951 | 0 | 11,48,426 | 5,14,425 | 248 | 1,05,797 | 0 | 1,964 | 5,16,575 | 23,14,386 |
| **Extraction** | 3,62,697 | 0 | 80,62,024 | 36,76,860 | 16,958 | 4,25,138 | 0 | 19,331 | 9,32,690 | 1,34,95,698 |
| **Osmf/leukoplakia** | 18,155 | 0 | 68,241 | 92,304 | 2,894 | 22,611 | 681 | 1,047 | 28,108 | 2,34,041 |
| **Toothache** | 1,36,125 | 296 | 15,07,034 | 9,24,318 | 11,550 | 60,354 | 0 | 1,062 | 3,15,064 | 29,55,803 |
| **Discoloration tooth** | 56,530 | 0 | 2,70,356 | 78,512 | 228 | 10,690 | 0 | 0 | 25,770 | 4,42,086 |
| **Loss of teeth** | 5,440 | 0 | 1,37,063 | 36,797 | 248 | 4,498 | 0 | 0 | 2,506 | 1,86,552 |
| **Others** | 1,28,938 | 832 | 8,22,542 | 7,74,019 | 5,798 | 1,12,364 | 145 | 9,981 | 2,23,064 | 20,77,683 |
| **Total** | **43,45,775** | **3,048** | **6,55,24,003** | **3,26,26,556** | **1,95,952** | **39,46,464** |  | **91,038** | **83,70,723** | **11,51,04,511** |
